# Statistical tests, *P* values, confidence intervals, and power: a guide to misinterpretations

**DOI:** 10.1007/s10654-016-0149-3

**Published:** 2016-05-21

**Authors:** Sander Greenland, Stephen J. Senn, Kenneth J. Rothman, John B. Carlin, Charles Poole, Steven N. Goodman, Douglas G. Altman

**Affiliations:** Department of Epidemiology and Department of Statistics, University of California, Los Angeles, CA USA; Competence Center for Methodology and Statistics, Luxembourg Institute of Health, Strassen, Luxembourg; RTI Health Solutions, Research Triangle Institute, Research Triangle Park, NC USA; Clinical Epidemiology and Biostatistics Unit, Murdoch Children’s Research Institute, School of Population Health, University of Melbourne, Melbourne, VIC Australia; Department of Epidemiology, Gillings School of Global Public Health, University of North Carolina, Chapel Hill, NC USA; Meta-Research Innovation Center, Departments of Medicine and of Health Research and Policy, Stanford University School of Medicine, Stanford, CA USA; Centre for Statistics in Medicine, Nuffield Department of Orthopaedics, Rheumatology and Musculoskeletal Sciences, University of Oxford, Oxford, UK

**Keywords:** Confidence intervals, Hypothesis testing, Null testing, *P* value, Power, Significance tests, Statistical testing

## Abstract

Misinterpretation and abuse of statistical tests, confidence intervals, and statistical power have been decried for decades, yet remain rampant. A key problem is that there are no interpretations of these concepts that are at once simple, intuitive, correct, and foolproof. Instead, correct use and interpretation of these statistics requires an attention to detail which seems to tax the patience of working scientists. This high cognitive demand has led to an epidemic of shortcut definitions and interpretations that are simply wrong, sometimes disastrously so—and yet these misinterpretations dominate much of the scientific literature. In light of this problem, we provide definitions and a discussion of basic statistics that are more general and critical than typically found in traditional introductory expositions. Our goal is to provide a resource for instructors, researchers, and consumers of statistics whose knowledge of statistical theory and technique may be limited but who wish to avoid and spot misinterpretations. We emphasize how violation of often unstated analysis protocols (such as selecting analyses for presentation based on the *P* values they produce) can lead to small *P* values even if the declared test hypothesis is correct, and can lead to large *P* values even if that hypothesis is incorrect. We then provide an explanatory list of 25 misinterpretations of *P* values, confidence intervals, and power. We conclude with guidelines for improving statistical interpretation and reporting.

## Introduction

Misinterpretation and abuse of statistical tests has been decried for decades, yet remains so rampant that some scientific journals discourage use of “statistical significance” (classifying results as “significant” or not based on a *P* value) [1]. One journal now bans all statistical tests and mathematically related procedures such as confidence intervals [2], which has led to considerable discussion and debate about the merits of such bans [3, 4].

Despite such bans, we expect that the statistical methods at issue will be with us for many years to come. We thus think it imperative that basic teaching as well as general understanding of these methods be improved. Toward that end, we attempt to explain the meaning of significance tests, confidence intervals, and statistical power in a more general and critical way than is traditionally done, and then review 25 common misconceptions in light of our explanations. We also discuss a few more subtle but nonetheless pervasive problems, explaining why it is important to examine and synthesize all results relating to a scientific question, rather than focus on individual findings. We further explain why statistical tests should never constitute the sole input to inferences or decisions about associations or effects. Among the many reasons are that, in most scientific settings, the arbitrary classification of results into “significant” and “non-significant” is unnecessary for and often damaging to valid interpretation of data; and that estimation of the size of effects and the uncertainty surrounding our estimates will be far more important for scientific inference and sound judgment than any such classification.

More detailed discussion of the general issues can be found in many articles, chapters, and books on statistical methods and their interpretation [5–20]. Specific issues are covered at length in these sources and in the many peer-reviewed articles that critique common misinterpretations of null-hypothesis testing and “statistical significance” [1, 12, 21–74].

## Statistical tests, *P* values, and confidence intervals: a caustic primer

### Statistical models, hypotheses, and tests

Every method of statistical inference depends on a complex web of assumptions about how data were collected and analyzed, and how the analysis results were selected for presentation. The full set of assumptions is embodied in a *statistical model* that underpins the method. This model is a mathematical representation of data variability, and thus ideally would capture accurately all sources of such variability. Many problems arise however because this statistical model often incorporates unrealistic or at best unjustified assumptions. This is true even for so-called “non-parametric” methods, which (like other methods) depend on assumptions of random sampling or randomization. These assumptions are often deceptively simple to write down mathematically, yet in practice are difficult to satisfy and verify, as they may depend on successful completion of a long sequence of actions (such as identifying, contacting, obtaining consent from, obtaining cooperation of, and following up subjects, as well as adherence to study protocols for treatment allocation, masking, and data analysis).

There is also a serious problem of defining the scope of a model, in that it should allow not only for a good representation of the observed data but also of hypothetical alternative data that might have been observed. The reference frame for data that “might have been observed” is often unclear, for example if multiple outcome measures or multiple predictive factors have been measured, and many decisions surrounding analysis choices have been made after the data were collected—as is invariably the case [33].

The difficulty of understanding and assessing underlying assumptions is exacerbated by the fact that the statistical model is usually presented in a highly compressed and abstract form—if presented at all. As a result, many assumptions go unremarked and are often unrecognized by users as well as consumers of statistics. Nonetheless, all statistical methods and interpretations are premised on the model assumptions; that is, on an assumption that the model provides a valid representation of the variation we would expect to see across data sets, faithfully reflecting the circumstances surrounding the study and phenomena occurring within it.

In most applications of statistical testing, one assumption in the model is a hypothesis that a particular effect has a specific size, and has been targeted for statistical analysis. (For simplicity, we use the word “effect” when “association or effect” would arguably be better in allowing for noncausal studies such as most surveys.) This targeted assumption is called the study hypothesis or *test hypothesis*, and the statistical methods used to evaluate it are called *statistical hypothesis tests*. Most often, the targeted effect size is a “null” value representing *zero* effect (e.g., that the study treatment makes no difference in average outcome), in which case the test hypothesis is called the *null hypothesis*. Nonetheless, it is also possible to test other effect sizes. We may also test hypotheses that the effect does or does not fall within a specific range; for example, we may test the hypothesis that the effect is no greater than a particular amount, in which case the hypothesis is said to be a *one*-*sided* or *dividing* hypothesis [7, 8].

Much statistical teaching and practice has developed a strong (and unhealthy) focus on the idea that the main aim of a study should be to test null hypotheses. In fact most descriptions of statistical testing focus *only* on testing null hypotheses, and the entire topic has been called “Null Hypothesis Significance Testing” (NHST). This exclusive focus on null hypotheses contributes to misunderstanding of tests. Adding to the misunderstanding is that many authors (including R.A. Fisher) use “null hypothesis” to refer to any test hypothesis, even though this usage is at odds with other authors and with ordinary English definitions of “null”—as are statistical usages of “significance” and “confidence.”

### Uncertainty, probability, and statistical significance

A more refined goal of statistical analysis is to provide an evaluation of certainty or uncertainty regarding the size of an effect. It is natural to express such certainty in terms of “probabilities” of hypotheses. In conventional statistical methods, however, “probability” refers not to hypotheses, but to quantities that are hypothetical frequencies of data patterns under an assumed statistical model. These methods are thus called *frequentist* methods, and the hypothetical frequencies they predict are called “frequency probabilities.” Despite considerable training to the contrary, many statistically educated scientists revert to the habit of misinterpreting these frequency probabilities as hypothesis probabilities. (Even more confusingly, the term “likelihood of a parameter value” is reserved by statisticians to refer to the probability of the observed data *given* the parameter value; it does not refer to a probability of the parameter taking on the given value.)

Nowhere are these problems more rampant than in applications of a hypothetical frequency called the *P* value, also known as the “observed significance level” for the test hypothesis. Statistical “significance tests” based on this concept have been a central part of statistical analyses for centuries [75]. The focus of traditional definitions of *P* values and statistical significance has been on null hypotheses, treating all other assumptions used to compute the *P* value as if they were known to be correct. Recognizing that these other assumptions are often questionable if not unwarranted, we will adopt a more general view of the *P* value as a statistical summary of the compatibility between the observed data and what we would predict or expect to see if we knew the entire statistical model (*all* the assumptions used to compute the *P* value) were correct.

Specifically, the distance between the data and the model prediction is measured using a *test statistic* (such as a t-statistic or a Chi squared statistic). The *P* value is then the probability that the chosen test statistic would have been *at least* as large as its observed value if *every* model assumption were correct, including the test hypothesis. This definition embodies a crucial point lost in traditional definitions: In logical terms, the *P* value tests *all* the assumptions about how the data were generated (the entire model), not just the targeted hypothesis it is supposed to test (such as a null hypothesis). Furthermore, these assumptions include far more than what are traditionally presented as modeling or probability assumptions—they include assumptions about the conduct of the analysis, for example that intermediate analysis results were not used to determine which analyses would be presented.

It is true that the smaller the *P* value, the more unusual the data would be *if* every single assumption were correct; but a very small *P* value does *not* tell us which assumption is incorrect. For example, the *P* value may be very small because the targeted hypothesis is false; but it may instead (or in addition) be very small because the study protocols were violated, or because it was selected for presentation based on its small size. Conversely, a large *P* value indicates only that the data are not unusual under the model, but does not imply that the model or any aspect of it (such as the targeted hypothesis) is correct; it may instead (or in addition) be large because (again) the study protocols were violated, or because it was selected for presentation based on its large size.

The general definition of a *P* value may help one to understand why statistical tests tell us much less than what many think they do: Not only does a *P* value *not* tell us whether the hypothesis targeted for testing is true or not; it says nothing specifically related to that hypothesis unless we can be completely assured that every other assumption used for its computation is correct—an assurance that is lacking in far too many studies.

Nonetheless, the *P* value can be viewed as a continuous measure of the compatibility between the data and the entire model used to compute it, ranging from 0 for complete incompatibility to 1 for perfect compatibility, and in this sense may be viewed as measuring the fit of the model to the data. Too often, however, the *P* value is degraded into a dichotomy in which results are declared “statistically significant” if *P* falls on or below a cut-off (usually 0.05) and declared “nonsignificant” otherwise. The terms “significance level” and “alpha level” (α) are often used to refer to the cut-off; however, the term “significance level” invites confusion of the cut-off with the *P* value itself. Their difference is profound: the cut-off value α is supposed to be fixed in advance and is thus part of the study design, unchanged in light of the data. In contrast, the *P* value is a number computed from the data and thus an analysis result, unknown until it is computed.

### Moving from tests to estimates

We can vary the test hypothesis while leaving other assumptions unchanged, to see how the *P* value differs across competing test hypotheses. Usually, these test hypotheses specify different sizes for a targeted effect; for example, we may test the hypothesis that the average difference between two treatment groups is zero (the null hypothesis), or that it is 20 or −10 or any size of interest. The effect size whose test produced *P* = 1 is the size most compatible with the data (in the sense of predicting what was in fact observed) *if* all the other assumptions used in the test (the statistical model) were correct, and provides a *point estimate* of the effect under those assumptions. The effect sizes whose test produced *P* > 0.05 will typically define a range of sizes (e.g., from 11.0 to 19.5) that would be considered more compatible with the data (in the sense of the observations being closer to what the model predicted) than sizes outside the range—again, *if* the statistical model were correct. This range corresponds to a 1 − 0.05 = 0.95 or 95 % *confidence interval*, and provides a convenient way of summarizing the results of hypothesis tests for many effect sizes. Confidence intervals are examples of *interval estimates*.

Neyman [76] proposed the construction of confidence intervals in this way because they have the following property: If one calculates, say, 95 % confidence intervals repeatedly *in valid applications*, 95 % of them, on average, will contain (i.e., include or cover) the true effect size. Hence, the specified confidence level is called the *coverage probability.* As Neyman stressed repeatedly, this coverage probability is a property of a long sequence of confidence intervals computed from valid models, rather than a property of any single confidence interval.

Many journals now require confidence intervals, but most textbooks and studies discuss *P* values only for the null hypothesis of no effect. This exclusive focus on null hypotheses in testing not only contributes to misunderstanding of tests and underappreciation of estimation, but also obscures the close relationship between *P* values and confidence intervals, as well as the weaknesses they share.

## What *P* values, confidence intervals, and power calculations don’t tell us

Much distortion arises from basic misunderstanding of what *P* values and their relatives (such as confidence intervals) do *not* tell us. Therefore, based on the articles in our reference list, we review prevalent *P* value misinterpretations as a way of moving toward defensible interpretations and presentations. We adopt the format of Goodman [40] in providing a list of misinterpretations that can be used to critically evaluate conclusions offered by research reports and reviews. Every one of the bolded statements in our list has contributed to statistical distortion of the scientific literature, and we add the emphatic “No!” to underscore statements that are not only fallacious but also not “true enough for practical purposes.”

### Common misinterpretations of single *P* values

1. **The*P*value is the probability that the test hypothesis is true; for example, if a test of the null hypothesis gave*P*** **=** **0.01, the null hypothesis has only a 1** **% chance of being true; if instead it gave*P*** **=** **0.40, the null hypothesis has a 40** **% chance of being true**. No! The *P* value *assumes* the test hypothesis is true—it is *not* a hypothesis probability and may be far from any reasonable probability for the test hypothesis. The *P* value simply indicates the degree to which the data conform to the pattern predicted by the test hypothesis and all the other assumptions used in the test (the underlying statistical model). Thus *P* = 0.01 would indicate that the data are not very close to what the statistical model (including the test hypothesis) predicted they should be, while *P* = 0.40 would indicate that the data are much closer to the model prediction, allowing for chance variation.
2. **The*P*value for the null hypothesis is the probability that chance alone produced the observed association; for example, if the*P*value for the null hypothesis is 0.08, there is an 8** **% probability that chance alone produced the association.** No! This is a common variation of the first fallacy and it is just as false. To say that chance *alone* produced the observed association is logically equivalent to asserting that every assumption used to compute the *P* value is correct, *including the null hypothesis*. Thus to claim that the null *P* value is the probability that chance alone produced the observed association is completely backwards: The *P* value is a probability computed *assuming* chance was operating alone. The absurdity of the common backwards interpretation might be appreciated by pondering how the *P* value, which is a probability deduced *from* a set of assumptions (the statistical model), can possibly refer to the probability *of* those assumptions.
  **Note**: One often sees “alone” dropped from this description (becoming “the *P* value for the null hypothesis is the probability that chance produced the observed association”), so that the statement is more ambiguous, but just as wrong.
3. **A significant test result (*P*** **≤** **0.05) means that the test hypothesis is false or should be rejected**. No! A small *P* value simply flags the data as being unusual if all the assumptions used to compute it (including the test hypothesis) were correct; it may be small because there was a large random error or because some assumption other than the test hypothesis was violated (for example, the assumption that this *P* value was not selected for presentation because it was below 0.05). *P* ≤ 0.05 only means that a discrepancy from the hypothesis prediction (e.g., no difference between treatment groups) would be as large *or larger than* that observed no more than 5 % of the time if *only* chance were creating the discrepancy (as opposed to a violation of the test hypothesis or a mistaken assumption).
4. **A nonsignificant test result (*P*** **>** **0.05) means that the test hypothesis is true or should be accepted**. No! A large *P* value only suggests that the data are *not* unusual if all the assumptions used to compute the *P* value (including the test hypothesis) were correct. The same data would also not be unusual under many other hypotheses. Furthermore, even if the test hypothesis is wrong, the *P* value may be large because it was inflated by a large random error or because of some other erroneous assumption (for example, the assumption that this *P* value was not selected for presentation because it was above 0.05). *P* > 0.05 only means that a discrepancy from the hypothesis prediction (e.g., no difference between treatment groups) would be as large *or largerthan* that observed more than 5 % of the time if *only* chance were creating the discrepancy.
5. **A large*P*value is evidence in favor of the test hypothesis**. No! In fact, any *P* value less than 1 implies that the test hypothesis is *not* the hypothesis most compatible with the data, because any other hypothesis with a larger *P* value would be even more compatible with the data. A *P* value cannot be said to favor the test hypothesis except in relation to those hypotheses with smaller *P* values. Furthermore, a large *P* value often indicates only that the data are incapable of discriminating among many competing hypotheses (as would be seen immediately by examining the range of the confidence interval). For example, many authors will misinterpret *P* = 0.70 from a test of the null hypothesis as evidence for no effect, when in fact it indicates that, even though the null hypothesis is compatible with the data under the assumptions used to compute the *P* value, it is *not* the hypothesis most compatible with the data—that honor would belong to a hypothesis with *P* = 1. But even if *P* = 1, there will be many other hypotheses that are highly consistent with the data, so that a definitive conclusion of “no association” cannot be deduced from a *P* value, no matter how large.
6. **A null-hypothesis*P*value greater than 0.05 means that no effect was observed, or that absence of an effect was shown or demonstrated**. No! Observing *P* > 0.05 for the null hypothesis only means that the null is one among the many hypotheses that have *P* > 0.05. Thus, unless the point estimate (observed association) equals the null value exactly, it is a mistake to conclude from *P* > 0.05 that a study found “no association” or “no evidence” of an effect. If the null *P* value is less than 1 some association must be present in the data, and one must look at the point estimate to determine the effect size most compatible with the data under the assumed model.
7. **Statistical significance indicates a scientifically or substantively important relation has been detected**. No! Especially when a study is large, very minor effects or small assumption violations can lead to statistically significant tests of the null hypothesis. Again, a small null *P* value simply flags the data as being unusual if all the assumptions used to compute it (including the null hypothesis) were correct; but the way the data are unusual might be of no clinical interest. One must look at the confidence interval to determine which effect sizes of scientific or other substantive (e.g., clinical) importance are relatively compatible with the data, given the model.
8. **Lack of statistical significance indicates that the effect size is small**. No! Especially when a study is small, even large effects may be “drowned in noise” and thus fail to be detected as statistically significant by a statistical test. A large null *P* value simply flags the data as *not* being unusual if all the assumptions used to compute it (including the test hypothesis) were correct; but the same data will also not be unusual under many other models and hypotheses besides the null. Again, one must look at the confidence interval to determine whether it includes effect sizes of importance.
9. **The*P*value is the chance of our data occurring if the test hypothesis is true; for example,*P*** **=** **0.05 means that the observed association would occur only 5** **% of the time under the test hypothesis**. No! The *P* value refers not only to what we observed, but also observations *more extreme* than what we observed (where “extremity” is measured in a particular way). And again, the *P* value refers to a data frequency when all the assumptions used to compute it are correct. In addition to the test hypothesis, these assumptions include randomness in sampling, treatment assignment, loss, and missingness, as well as an assumption that the *P* value was not selected for presentation based on its size or some other aspect of the results.
10. **If you reject the test hypothesis because*P*** **≤** **0.05, the chance you are in error (the chance your “significant finding” is a false positive) is 5** **%**. No! To see why this description is false, suppose the test hypothesis is in fact true. Then, if you reject it, the chance you are in error is 100 %, not 5 %. The 5 % refers only to how often you would reject it, and therefore be in error, over very many uses of the test across different studies when the test hypothesis and all other assumptions used for the test are true. It does not refer to your single use of the test, which may have been thrown off by assumption violations as well as random errors. This is yet another version of misinterpretation #1.
11. ***P*** **=** **0.05 and*P*** **≤** **0.05 mean the same thing**. No! This is like saying reported height = 2 m and reported height ≤2 m are the same thing: “height = 2 m” would include few people and those people would be considered tall, whereas “height ≤2 m” would include most people including small children. Similarly, *P* = 0.05 would be considered a borderline result in terms of statistical significance, whereas *P* ≤ 0.05 lumps borderline results together with results very incompatible with the model (e.g., *P* = 0.0001) thus rendering its meaning vague, for no good purpose.
12. ***P*values are properly reported as inequalities (e.g., report “*P*** **<** **0.02” when*P*** **=** **0.015 or report “*P*** **>** **0.05” when*P*** **=** **0.06 or*P*** **=** **0.70)**. No! This is bad practice because it makes it difficult or impossible for the reader to accurately interpret the statistical result. Only when the *P* value is very small (e.g., under 0.001) does an inequality become justifiable: There is little practical difference among very small *P* values when the assumptions used to compute *P* values are not known with enough certainty to justify such precision, and most methods for computing *P* values are not numerically accurate below a certain point.
13. **Statistical significance is a property of the phenomenon being studied, and thus statistical tests detect significance**. No! This misinterpretation is promoted when researchers state that they have or have not found “evidence of” a statistically significant effect. The effect being tested either exists or does not exist. “Statistical significance” is a dichotomous description of a *P* value (that it is below the chosen cut-off) and thus is a property of a result of a statistical test; it is not a property of the effect or population being studied.
14. **One should always use two-sided*P*values**. No! Two-sided *P* values are designed to test hypotheses that the targeted effect measure equals a specific value (e.g., zero), and is neither above nor below this value. When, however, the test hypothesis of scientific or practical interest is a one-sided (dividing) hypothesis, a one-sided *P* value is appropriate. For example, consider the practical question of whether a new drug is *at least* as good as the standard drug for increasing survival time. This question is one-sided, so testing this hypothesis calls for a one-sided *P* value. Nonetheless, because two-sided *P* values are the usual default, it will be important to note when and why a one-sided *P* value is being used instead.

There are other interpretations of *P* values that are controversial, in that whether a categorical “No!” is warranted depends on one’s philosophy of statistics and the precise meaning given to the terms involved. The disputed claims deserve recognition if one wishes to avoid such controversy.

For example, it has been argued that *P* values overstate evidence against test hypotheses, based on directly comparing *P* values against certain quantities (likelihood ratios and Bayes factors) that play a central role as evidence measures in Bayesian analysis [37, 72, 77–83]. Nonetheless, many other statisticians do not accept these quantities as gold standards, and instead point out that *P* values summarize crucial evidence needed to gauge the error rates of decisions based on statistical tests (even though they are far from sufficient for making those decisions). Thus, from this frequentist perspective, *P* values do not overstate evidence and may even be considered as measuring one aspect of evidence [7, 8, 84–87], with 1 − *P* measuring evidence against the model used to compute the *P* value. See also Murtaugh [88] and its accompanying discussion.

### Common misinterpretations of *P* value comparisons and predictions

Some of the most severe distortions of the scientific literature produced by statistical testing involve erroneous comparison and synthesis of results from different studies or study subgroups. Among the worst are:

15. **When the same hypothesis is tested in different studies and none or a minority of the tests are statistically significant (all*P*** **>** **0.05), the overall evidence supports the hypothesis**. No! This belief is often used to claim that a literature supports no effect when the opposite is case. It reflects a tendency of researchers to “overestimate the power of most research” [89]. In reality, every study could fail to reach statistical significance and yet when combined show a statistically significant association and persuasive evidence of an effect. For example, if there were five studies each with *P* = 0.10, none would be significant at 0.05 level; but when these *P* values are combined using the Fisher formula [9], the overall *P* value would be 0.01. There are many real examples of persuasive evidence for important effects when few studies or even no study reported “statistically significant” associations [90, 91]. Thus, lack of statistical significance of individual studies should not be taken as implying that the totality of evidence supports no effect.
16. **When the same hypothesis is tested in two different populations and the resulting*P*values are on opposite sides of 0.05, the results are conflicting**. No! Statistical tests are sensitive to many differences between study populations that are irrelevant to whether their results are in agreement, such as the sizes of compared groups in each population. As a consequence, two studies may provide very different *P* values for the same test hypothesis and yet be in perfect agreement (e.g., may show identical observed associations). For example, suppose we had two randomized trials A and B of a treatment, identical except that trial A had a known standard error of 2 for the mean difference between treatment groups whereas trial B had a known standard error of 1 for the difference. If both trials observed a difference between treatment groups of exactly 3, the usual normal test would produce *P* = 0.13 in A but *P* = 0.003 in B. Despite their difference in *P* values, the test of the hypothesis of no difference in effect across studies would have *P* = 1, reflecting the perfect agreement of the observed mean differences from the studies. Differences between results must be evaluated by directly, for example by estimating and testing those differences to produce a confidence interval and a *P* value comparing the results (often called analysis of heterogeneity, interaction, or modification).
17. **When the same hypothesis is tested in two different populations and the same*P*values are obtained, the results are in agreement**. No! Again, tests are sensitive to many differences between populations that are irrelevant to whether their results are in agreement. Two different studies may even exhibit identical *P* values for testing the same hypothesis yet also exhibit clearly different observed associations. For example, suppose randomized experiment A observed a mean difference between treatment groups of 3.00 with standard error 1.00, while B observed a mean difference of 12.00 with standard error 4.00. Then the standard normal test would produce *P* = 0.003 in both; yet the test of the hypothesis of no difference in effect across studies gives *P* = 0.03, reflecting the large difference (12.00 − 3.00 = 9.00) between the mean differences.
18. **If one observes a small*P*value, there is a good chance that the next study will produce a*P*value at least as small for the same hypothesis.** No! This is false even under the ideal condition that both studies are independent and all assumptions including the test hypothesis are correct in both studies. In that case, if (say) one observes *P* = 0.03, the chance that the new study will show *P* ≤ 0.03 is only 3 %; thus the chance the new study will show a *P* value as small or smaller (the “replication probability”) is exactly the observed *P* value! If on the other hand the small *P* value arose solely because the true effect exactly equaled its observed estimate, there would be a 50 % chance that a repeat experiment of identical design would have a larger *P* value [37]. In general, the size of the new *P* value will be extremely sensitive to the study size and the extent to which the test hypothesis or other assumptions are violated in the new study [86]; in particular, *P* may be very small or very large depending on whether the study and the violations are large or small.

Finally, although it is (we hope obviously) wrong to do so, one sometimes sees the null hypothesis compared with another (alternative) hypothesis using a two-sided *P* value for the null and a one-sided *P* value for the alternative. This comparison is biased in favor of the null in that the two-sided test will falsely reject the null only half as often as the one-sided test will falsely reject the alternative (again, under all the assumptions used for testing).

### Common misinterpretations of confidence intervals

Most of the above misinterpretations translate into an analogous misinterpretation for confidence intervals. For example, another misinterpretation of *P* > 0.05 is that it means the test hypothesis has only a 5 % chance of being false, which in terms of a confidence interval becomes the common fallacy:

19. **The specific 95** **% confidence interval presented by a study has a 95** **% chance of containing the true effect size**. No! A reported confidence interval is a range between two numbers. The frequency with which an observed interval (e.g., 0.72–2.88) contains the true effect is either 100 % if the true effect is within the interval or 0 % if not; the 95 % refers only to how often 95 % confidence intervals computed from very many studies would contain the true size *if all the assumptions used to compute the intervals were correct*. It is possible to compute an interval that can be interpreted as having 95 % probability of containing the true value; nonetheless, such computations require not only the assumptions used to compute the confidence interval, but also further assumptions about the size of effects in the model. These further assumptions are summarized in what is called a *prior distribution*, and the resulting intervals are usually called *Bayesian posterior (or credible) intervals* to distinguish them from confidence intervals [18].

Symmetrically, the misinterpretation of a small *P* value as disproving the test hypothesis could be translated into:

20. **An effect size outside the 95** **% confidence interval has been refuted (or excluded) by the data**. No! As with the *P* value, the confidence interval is computed from many assumptions, the violation of which may have led to the results. Thus it is the combination of the data with the assumptions, along with the arbitrary 95 % criterion, that are needed to declare an effect size outside the interval is in some way incompatible with the observations. Even then, judgements as extreme as saying the effect size has been refuted or excluded will require even stronger conditions.

As with *P* values, naïve comparison of confidence intervals can be highly misleading:

21. **If two confidence intervals overlap, the difference between two estimates or studies is not significant**. No! The 95 % confidence intervals from two subgroups or studies may overlap substantially and yet the test for difference between them may still produce *P* < 0.05. Suppose for example, two 95 % confidence intervals for means from normal populations with known variances are (1.04, 4.96) and (4.16, 19.84); these intervals overlap, yet the test of the hypothesis of no difference in effect across studies gives *P* = 0.03. As with *P* values, comparison between groups requires statistics that directly test and estimate the differences across groups. It can, however, be noted that if the two 95 % confidence intervals fail to overlap, then when using the same assumptions used to compute the confidence intervals we will find *P* < 0.05 for the difference; and if one of the 95 % intervals contains the point estimate from the other group or study, we will find *P* > 0.05 for the difference.

Finally, as with *P* values, the replication properties of confidence intervals are usually misunderstood:

22. **An observed 95** **% confidence interval predicts that 95** **% of the estimates from future studies will fall inside the observed interval.** No! This statement is wrong in several ways. Most importantly, under the model, 95 % is the frequency with which *otherunobserved* intervals will contain the *true effect*, not how frequently the one interval being presented will contain future estimates. In fact, even under ideal conditions the chance that a future estimate will fall within the current interval will usually be much less than 95 %. For example, if two independent studies of the same quantity provide unbiased normal point estimates with the same standard errors, the chance that the 95 % confidence interval for the first study contains the point estimate from the second is 83 % (which is the chance that the difference between the two estimates is less than 1.96 standard errors). Again, an observed interval either does or does not contain the true effect; the 95 % refers only to how often 95 % confidence intervals computed from very many studies would contain the true effect if all the assumptions used to compute the intervals were correct.
23. **If one 95** **% confidence interval includes the null value and another excludes that value, the interval excluding the null is the more precise one**. No! When the model is correct, precision of statistical estimation is measured directly by confidence interval *width* (measured on the appropriate scale). It is not a matter of inclusion or exclusion of the null or any other value. Consider two 95 % confidence intervals for a difference in means, one with limits of 5 and 40, the other with limits of −5 and 10. The first interval excludes the null value of 0, but is 30 units wide. The second includes the null value, but is half as wide and therefore much more precise.

In addition to the above misinterpretations, 95 % confidence intervals force the 0.05-level cutoff on the reader, lumping together all effect sizes with *P* > 0.05, and in this way are as bad as presenting *P* values as dichotomies. Nonetheless, many authors agree that confidence intervals are superior to tests and *P* values because they allow one to shift focus away from the null hypothesis, toward the full range of effect sizes compatible with the data—a shift recommended by many authors and a growing number of journals. Another way to bring attention to non-null hypotheses is to present their *P* values; for example, one could provide or demand *P* values for those effect sizes that are recognized as scientifically reasonable alternatives to the null.

As with *P* values, further cautions are needed to avoid misinterpreting confidence intervals as providing sharp answers when none are warranted. The hypothesis which says the point estimate is the correct effect will have the largest *P* value (*P* = 1 in most cases), and hypotheses inside a confidence interval will have higher *P* values than hypotheses outside the interval. The *P* values will vary greatly, however, among hypotheses inside the interval, as well as among hypotheses on the outside. Also, two hypotheses may have nearly equal *P* values even though one of the hypotheses is inside the interval and the other is outside. Thus, if we use *P* values to measure compatibility of hypotheses with data and wish to compare hypotheses with this measure, we need to examine their *P* values directly, not simply ask whether the hypotheses are inside or outside the interval. This need is particularly acute when (as usual) one of the hypotheses under scrutiny is a null hypothesis.

### Common misinterpretations of power

The *power* of a test to detect a correct alternative hypothesis is the pre-study probability that the test will reject the test hypothesis (e.g., the probability that *P* will not exceed a pre-specified cut-off such as 0.05). (The corresponding pre-study probability of failing to reject the test hypothesis when the alternative is correct is one minus the power, also known as the Type-II or beta error rate) [84] As with *P* values and confidence intervals, this probability is defined over repetitions of the same study design and so is a frequency probability. One source of reasonable alternative hypotheses are the effect sizes that were used to compute power in the study proposal. Pre-study power calculations do not, however, measure the compatibility of these alternatives with the data actually observed, while power calculated from the observed data is a direct (if obscure) transformation of the null *P* value and so provides no test of the alternatives. Thus, presentation of power does not obviate the need to provide interval estimates and direct tests of the alternatives.

For these reasons, many authors have condemned use of power to interpret estimates and statistical tests [42, 92–97], arguing that (in contrast to confidence intervals) it distracts attention from direct comparisons of hypotheses and introduces new misinterpretations, such as:

24. **If you accept the null hypothesis because the null*P*value exceeds 0.05 and the power of your test is 90** **%, the chance you are in error (the chance that your finding is a false negative) is 10** **%**. No! If the null hypothesis is false and you accept it, the chance you are in error is 100 %, not 10 %. Conversely, if the null hypothesis is true and you accept it, the chance you are in error is 0 %. The 10 % refers only to how often you would be in error over very many uses of the test across different studies when the particular alternative used to compute power is correct *and* all other assumptions used for the test are correct in all the studies. It does not refer to your single use of the test or your error rate under any alternative effect size other than the one used to compute power.

It can be especially misleading to compare results for two hypotheses by presenting a test or *P* value for one and power for the other. For example, testing the null by seeing whether *P* ≤ 0.05 with a power less than 1 − 0.05 = 0.95 for the alternative (as done routinely) will bias the comparison in favor of the null because it entails a lower probability of incorrectly rejecting the null (0.05) than of incorrectly accepting the null when the alternative is correct. Thus, claims about relative support or evidence need to be based on direct and comparable measures of support or evidence for both hypotheses, otherwise mistakes like the following will occur:

25. **If the null*P*value exceeds 0.05 and the power of this test is 90** **% at an alternative, the results support the null over the alternative**. This claim seems intuitive to many, but counterexamples are easy to construct in which the null *P* value is between 0.05 and 0.10, and yet there are alternatives whose own *P* value exceeds 0.10 and for which the power is 0.90. Parallel results ensue for other accepted measures of compatibility, evidence, and support, indicating that the data show lower compatibility with and more evidence against the null than the alternative, despite the fact that the null *P* value is “not significant” at the 0.05 alpha level and the power against the alternative is “very high” [42].

Despite its shortcomings for interpreting current data, power can be useful for designing studies and for understanding why replication of “statistical significance” will often fail even under ideal conditions. Studies are often designed or claimed to have 80 % power against a key alternative when using a 0.05 significance level, although in execution often have less power due to unanticipated problems such as low subject recruitment. Thus, if the alternative is correct and the actual power of two studies is 80 %, the chance that the studies will both show *P* ≤ 0.05 will at best be only 0.80(0.80) = 64 %; furthermore, the chance that one study shows *P* ≤ 0.05 and the other does not (and thus will be misinterpreted as showing conflicting results) is 2(0.80)0.20 = 32 % or about 1 chance in 3. Similar calculations taking account of typical problems suggest that one could anticipate a “replication crisis” even if there were no publication or reporting bias, simply because current design and testing conventions treat individual study results as dichotomous outputs of “significant”/“nonsignificant” or “reject”/“accept.”

## A statistical model is much more than an equation with Greek letters

The above list could be expanded by reviewing the research literature. We will however now turn to direct discussion of an issue that has been receiving more attention of late, yet is still widely overlooked or interpreted too narrowly in statistical teaching and presentations: That the statistical model used to obtain the results is correct.

Too often, the full statistical model is treated as a simple regression or structural equation in which effects are represented by parameters denoted by Greek letters. “Model checking” is then limited to tests of fit or testing additional terms for the model. Yet these tests of fit themselves make further assumptions that should be seen as part of the full model. For example, all common tests and confidence intervals depend on assumptions of random selection for observation or treatment and random loss or missingness within levels of controlled covariates. These assumptions have gradually come under scrutiny via sensitivity and bias analysis [98], but such methods remain far removed from the basic statistical training given to most researchers.

Less often stated is the even more crucial assumption that the analyses themselves were not guided toward finding nonsignificance or significance (analysis bias), and that the analysis results were not reported based on their nonsignificance or significance (reporting bias and publication bias). Selective reporting renders false even the limited ideal meanings of statistical significance, *P* values, and confidence intervals. Because author decisions to report and editorial decisions to publish results often depend on whether the *P* value is above or below 0.05, selective reporting has been identified as a major problem in large segments of the scientific literature [99–101].

Although this selection problem has also been subject to sensitivity analysis, there has been a bias in studies of reporting and publication bias: It is usually assumed that these biases favor significance. This assumption is of course correct when (as is often the case) researchers select results for presentation when *P* ≤ 0.05, a practice that tends to exaggerate associations [101–105]. Nonetheless, bias in favor of reporting *P* ≤ 0.05 is not always plausible let alone supported by evidence or common sense. For example, one might expect selection for *P* > 0.05 in publications funded by those with stakes in acceptance of the null hypothesis (a practice which tends to understate associations); in accord with that expectation, some empirical studies have observed smaller estimates and “nonsignificance” more often in such publications than in other studies [101, 106, 107].

Addressing such problems would require far more political will and effort than addressing misinterpretation of statistics, such as enforcing registration of trials, along with open data and analysis code from all completed studies (as in the AllTrials initiative, http://www.alltrials.net/). In the meantime, readers are advised to consider the entire context in which research reports are produced and appear when interpreting the statistics and conclusions offered by the reports.

## Conclusions

Upon realizing that statistical tests are usually misinterpreted, one may wonder what if anything these tests do for science. They were originally intended to account for random variability as a source of error, thereby sounding a note of caution against overinterpretation of observed associations as true effects or as stronger evidence against null hypotheses than was warranted. But before long that use was turned on its head to provide fallacious support for null hypotheses in the form of “failure to achieve” or “failure to attain” statistical significance.

We have no doubt that the founders of modern statistical testing would be horrified by common treatments of their invention. In their first paper describing their binary approach to statistical testing, Neyman and Pearson [108] wrote that “it is doubtful whether the knowledge that [a *P* value] was really 0.03 (or 0.06), rather than 0.05…would in fact ever modify our judgment” and that “The tests themselves give no final verdict, but as tools help the worker who is using them to form his final decision.” Pearson [109] later added, “No doubt we could more aptly have said, ‘his final or provisional decision.’” Fisher [110] went further, saying “No scientific worker has a fixed level of significance at which from year to year, and in all circumstances, he rejects hypotheses; he rather gives his mind to each particular case in the light of his evidence and his ideas.” Yet fallacious and ritualistic use of tests continued to spread, including beliefs that whether *P* was above or below 0.05 was a universal arbiter of discovery. Thus by 1965, Hill [111] lamented that “too often we weaken our capacity to interpret data and to take reasonable decisions whatever the value of *P*. And far too often we deduce ‘no difference’ from ‘no significant difference.’”

In response, it has been argued that some misinterpretations are harmless in tightly controlled experiments on well-understood systems, where the test hypothesis may have special support from established theories (e.g., Mendelian genetics) and in which every other assumption (such as random allocation) is forced to hold by careful design and execution of the study. But it has long been asserted that the harms of statistical testing in more uncontrollable and amorphous research settings (such as social-science, health, and medical fields) have far outweighed its benefits, leading to calls for banning such tests in research reports—again with one journal banning *P* values as well as confidence intervals [2].

Given, however, the deep entrenchment of statistical testing, as well as the absence of generally accepted alternative methods, there have been many attempts to salvage *P* values by detaching them from their use in significance tests. One approach is to focus on *P* values as continuous measures of compatibility, as described earlier. Although this approach has its own limitations (as described in points 1, 2, 5, 9, 15, 18, 19), it avoids comparison of *P* values with arbitrary cutoffs such as 0.05, (as described in 3, 4, 6–8, 10–13, 15, 16, 21 and 23–25). Another approach is to teach and use correct relations of *P* values to hypothesis probabilities. For example, under common statistical models, one-sided *P* values can provide lower bounds on probabilities for hypotheses about effect directions [45, 46, 112, 113]. Whether such reinterpretations can eventually replace common misinterpretations to good effect remains to be seen.

A shift in emphasis from hypothesis testing to estimation has been promoted as a simple and relatively safe way to improve practice [5, 61, 63, 114, 115] resulting in increasing use of confidence intervals and editorial demands for them; nonetheless, this shift has brought to the fore misinterpretations of intervals such as 19–23 above [116]. Other approaches combine tests of the null with further calculations involving both null and alternative hypotheses [117, 118]; such calculations may, however, may bring with them further misinterpretations similar to those described above for power, as well as greater complexity.

Meanwhile, in the hopes of minimizing harms of current practice, we can offer several guidelines for users and readers of statistics, and re-emphasize some key warnings from our list of misinterpretations:

- Correct and careful interpretation of statistical tests demands examining the sizes of effect estimates and confidence limits, as well as precise *P* values (not just whether *P* values are above or below 0.05 or some other threshold).
- Careful interpretation also demands critical examination of the assumptions and conventions used for the statistical analysis—not just the usual statistical assumptions, but also the hidden assumptions about how results were generated and chosen for presentation.
- It is simply false to claim that statistically nonsignificant results support a test hypothesis, because the same results may be even more compatible with alternative hypotheses—even if the power of the test is high for those alternatives.
- Interval estimates aid in evaluating whether the data are capable of discriminating among various hypotheses about effect sizes, or whether statistical results have been misrepresented as supporting one hypothesis when those results are better explained by other hypotheses (see points 4–6). We caution however that confidence intervals are often only a first step in these tasks. To compare hypotheses in light of the data and the statistical model it may be necessary to calculate the *P* value (or relative likelihood) of each hypothesis. We further caution that confidence intervals provide only a best-case measure of the uncertainty or ambiguity left by the data, insofar as they depend on an uncertain statistical model.
- Correct statistical evaluation of multiple studies requires a pooled analysis or meta-analysis that deals correctly with study biases [68, 119–125]. Even when this is done, however, all the earlier cautions apply. Furthermore, the outcome of any statistical procedure is but one of many considerations that must be evaluated when examining the totality of evidence. In particular, statistical significance is neither necessary nor sufficient for determining the scientific or practical significance of a set of observations. This view was affirmed unanimously by the U.S. Supreme Court, (Matrixx Initiatives, Inc., et al. v. Siracusano et al. No. 09–1156. Argued January 10, 2011, Decided March 22, 2011), and can be seen in our earlier quotes from Neyman and Pearson.
- Any opinion offered about the *probability*, *likelihood*, *certainty*, or similar property for a hypothesis **cannot** be derived from statistical methods alone. In particular, significance tests and confidence intervals do not by themselves provide a logically sound basis for concluding an effect is present or absent with certainty or a given probability. This point should be borne in mind whenever one sees a conclusion framed as a statement of probability, likelihood, or certainty about a hypothesis. Information about the hypothesis beyond that contained in the analyzed data and in conventional statistical models (which give only data probabilities) must be used to reach such a conclusion; that information should be explicitly acknowledged and described by those offering the conclusion. Bayesian statistics offers methods that attempt to incorporate the needed information directly into the statistical model; they have not, however, achieved the popularity of *P* values and confidence intervals, in part because of philosophical objections and in part because no conventions have become established for their use.
- All statistical methods (whether frequentist or Bayesian, or for testing or estimation, or for inference or decision) make extensive assumptions about the sequence of events that led to the results presented—not only in the data generation, but in the analysis choices. Thus, to allow critical evaluation, research reports (including meta-analyses) should describe in detail the full sequence of events that led to the statistics presented, including the motivation for the study, its design, the original analysis plan, the criteria used to include and exclude subjects (or studies) and data, and a thorough description of all the analyses that were conducted.

In closing, we note that no statistical method is immune to misinterpretation and misuse, but prudent users of statistics will avoid approaches especially prone to serious abuse. In this regard, we join others in singling out the degradation of *P* values into “significant” and “nonsignificant” as an especially pernicious statistical practice [126].
